# Risk Stratification and Anticoagulation in Low-risk Non-valvular Atrial Fibrillation

**DOI:** 10.5812/cardiovascmed.8118

**Published:** 2013-02-24

**Authors:** Arash Arya

**Affiliations:** 1 Department of Electrophysiology, Heart Centre University of Leipzig, Leipzig, Germany

**Keywords:** Atrial Fibrillation, Risk, Anticioagulation

*Dear Editor,*

We thank Dr. Kiani for highlighting again the importance of risk stratification in patients with non-valvular atrial fibrillation (AF). We do agree with him as stated also in our review (see text and Figure 1) that the CHA2DS2-VASc score should be currently applied to patients with CHADS2-Score < 2 for further risk stratification as also recommended by current guidelines (1,2). It is noteworthy to mention that The CHA2DS2-VASc score has been validated in numerous AF populations and compared with CHADS2-Score (2). Therefore all patients with CHA2DS2-VASc score of 1 should be considered for stroke prevention, which is essentially treatment with oral anticoagulation agents. The HAS-BLED-Score (range 0-9, high risk ≥ 3) for bleeding can further help us to choose the appropriate oral anticoagulation agent in patients with non-valvular-AF (1,2). In patients with a CHADS2 score of 0 but with a high risk of bleeding, apixaban and dabigatran showed a net clinical benefit. In patients with a CHA2DS2-VASc score of 1, apixaban and both doses of dabigatran (150 and 110 mg twice daily) had a positive net clinical benefit. All three new oral anticoagulation agents, dabigatran, rivaroxaban, and apixaban, offer superior clinical benefit over warfarin in patients with a CHADS2 score ≥ 1 or CHA2DS2-VASc score ≥ 2, regardless of bleeding risk. When the risks of stroke and bleeding are both elevated, dabigatran, rivaroxaban, and apixaban appear to have a greater net clinical benefit than warfarin (2,3). As it is stated in our Review the Aspirin should not be used for stroke prevention in AF (1,2).

Finally, although clinically used in Europe, there is no conclusive evidence that left atrial appendage (LAA) occlusion reduces the risk of stroke in patients with non-valvular AF (4). Although the concept of LAA closure seems reasonable, the evidence on efficacy and safety is currently insufficient to recommend these approaches for any patients other than those in whom long-term OAC is absolutely contraindicated (Class IIb recommendation, Level of Evidence B) (4). Adequately powered, randomized studies comparing interventional LAA closure with oral anticoagulation (including new oral agents) are needed for adequate assessment of such techniques (4). It is worth to mention that the need for lifelong aspirin treatment after interventional LAA closure, and the significant bleeding risk with aspirin, may weigh against interventional LAA occlusion devices (5). Therefore although the LAA closure devices may be promising as stated by Dr. Kiani, few patients are suitable candidates for closure devices implantation especially in the era of new oral anticoagulation agents and currently very few selected patients at our center receive LAA closure devices.
